# Supplementary material for: Patterns of Intron Gain and Loss in Fungi
Source: PLoS Biol. 2004 Nov 30;2(12):e422. doi: 10.1371/journal.pbio.0020422 (PMC532390; doi:10.1371/journal.pbio.0020422)
Supplement: Table S1 — Also available at http://genes.mit.edu/NielsenEtAl/. (4.3 MB ZIP). [file pbio.0020422.st001.zip › NielsenEtAl/html/1413.html]

AN7698.1.NCU08067.1.MG09125.1.FG09435.1


```
 CLUSTAL W (1.82) Multiple Sequence Alignments - Introns Inserted


Sequence 1: AN7698.1	287 aa
Sequence 2: FG09435.1	302 aa
Sequence 3: NCU08067.1	304 aa
Sequence 4: MG09125.1	304 aa
Alignment Length: 326 aa
Number Identitical Residues: 124 aa
Alignment Score (without introns) 5829


MG09125.1 	MEHSRGQYGGGRKGMSLGNVIGDPFALATISIAG0LAWLIAFIASIVAQIQTTQG-----
NCU08067.1	MEHGRNSYR--RKGIDMGNIIGDPFALATTSIAT0LSWIIILFGSIFGFRDQNDGSNGAP
FG09435.1 	-------MYGQRKGIQMSNILGDPFALATLSIST0LAWFITFVSCIIGRIQQNGDDDLAK
AN7698.1  	-----------MAALRASNLLGDPFALATSSIAM0LGWLIAFIASIAADVQ---------
          	             .:  .*::******** **:  *.*:* :...* .  :         

MG09125.1 	---FPTYTWWTVVFYFFLIPGVFVVVASDTIQTYHVA~LVGYMACGLVLTTSSVNGLVYS
NCU08067.1	VIVWPTYSWFTLVFNFFLILGIFIVIASDSAQTYHVA~IVGYLAVGLVGSTSSINNLIYS
FG09435.1 	NDPFPTFVWWSCIYCLCLITGVFVVIGSDAVHTYHVA~VTGYLAAGIVLVSSSINQLLYS
AN7698.1  	-DPYPSFQWWAIAYSFCCNVGVIVVFLTDTGLTYGVA0VVGYLAASLVMNSISANSMFNS
          	   :*:: *::  : :    *:::*. :*:  ** ** :.**:* .:*  : * * :. *

MG09125.1 	TN-GAKEAAAAGFILLSMVT0IVWIFYFGSAPSAMPRAYLDSFALSKESTS----NNRQT
NCU08067.1	GV-ASMEATAAGYILLSMVT0IIWIFYFGSAPSAVPRAYIDSFALTKESTLPAHHMSRQT
FG09435.1 	NS-GAREAASAGFILLAMVI0VIWTFYFGSNPSSTPRAFLDSFALAKESTT----IHRST
AN7698.1  	KSTSSFQAAGAGFILLCMVN0IVWTFYFGSAPQAKHRGFIDSFALNKENQGS-YGANRPM
          	  :.: :*:.**:***.**  ::* ***** *.:  *.::***** **.  .     *  

MG09125.1 	MTGGGYGIG--------RPETSTS-VQPPQMYTSAQLNGFENPSPVNGMRNSGAPPSGFP
NCU08067.1	MNHNGLSSPNAYGSYNMRPETSASGLQPPQMYT-GQLNGLENP-----ARQSQIP-QGFS
FG09435.1 	MNAYG-----------GRPETSNS-VQPPQMYTSAQLNGFENPSPVGGIAGPRGSAVPQS
AN7698.1  	SSAYG-----------ARPETTTS-R--PQMYTSAQLNGFETSSPVSGYHG---GAPGET
          	 .  *            ****: *    *****:.****:*..:. ..       .   .

MG09125.1 	TTPGPASGLPKTTTPPAGGAADAEIVPPTEYPYRAKAIYTYEANPDDANEISFSKHEILE
NCU08067.1	SN-----NIPK----PQG---EGEIVPPTEYPYRAKAIFSYEANPDDANEISFSKHEVLE
FG09435.1 	YTNSGMQQQPPQSKPGHSPGNDTEVVPPTEYPYRAKAIYSYEANPDDANEISFSKHEILE
AN7698.1  	RSPSQARFTSLGGPNASNPDTVGEIPPPTEYPYKAKAIYKYEANPEDANEIGFEKGEELE
          	 . .     .       .     *: *******:****:.*****:*****.*.* * **

MG09125.1 	VSDVSGRWWQARKETGETGIAPSNYLILL
NCU08067.1	ISDVSGRWWQARKENGETGIAPSNYLILL
FG09435.1 	VSDVSGRWWQARKESGETGIAPSNYLILL
AN7698.1  	VSDVSGRWWQARKANGETGIAPSNYLILL
          	:************ .**************
```
